# The Bronchocele of New-born Infants

**Published:** 1850-12

**Authors:** 


### OBSTETRICS.

*The Bronchocele of New-born Infants.*—Dr. Betz, of Tübingen, has published an interesting essay upon this subject. He attributes the silence of authors respecting it rather to their having overlooked the affection than to its rarity. Such children are usually stout and full-blooded, and the enlarged thyroid may be mistaken for a mere fold of fatty integument. In some, the neck seems merely too broad, while in others it undergoes no change; these differences depending upon the part of the gland engaged.

Immediately, or very soon after birth, a marked difficulty of respiration comes on, which may prove fatal in a few hours, or in two or three days only. The inspirations are deep, being accompanied by a peculiar croaking tone, that may be heard outside the door. The expiration is also very laboured and sometimes accompanied by a cry. At times the breathing seems quite arrested, so that the child is in the most extreme danger from suffocation, until, with a cry, inspiration again occurs. The dyspnœa is sometimes irregularly paroxysmal. The alæ nasi are usually expanded, and the lips and hands of a blue color. Sucking is impossible, and attempts produce the most extreme dyspnœa, which is also excited if the child be fed, the greatest difficulty prevailing in getting it to swallow the least quantity. The mouth is full of saliva and mucus, which collect in small bladders between the lips.

According to the amount of disease, the remissions are longer or shorter, and the child sometimes at last goes off quite unexpectedly.

The affection consists in a simple hypertrophy of the thyroid ; no change in its normal structure, save perhaps some increase in its vascularity, being observable. The whole gland may be affected, giving a crescent shape, and, where an isthmus connects the two lobes, the neck assumes a great breadth. In other cases only one lobe, or even only the apex of that, may be affected, and the nature of the disease be undetected. The passage to the larynx and trachea is more or less impeded, while the posterior developments of the tumour impedes swallowing, and endangers suffocation in the attempt. The accumulation of mucus is an additional cause of obstruction. It is not merely the size of the swelling of the lobes, but its position, that determines the amount of danger.

The affection would seem sometimes to be hereditary, or, at all events, it affects several members of the same family. Various friends of Dr. Betz have observed this bronchocele of infants, but he is not disposed to consider it as especially endemic at Tübingen. He thinks that the suffocative dyspnœa, and death resulting from this disease, may elucidate the nature of some of the cases of laryngeal asthma, spasm of the glottis, thymic asthma, or laryngismus, concerning which so much confusion and doubt at present prevails. An enlargement of the thymus may certainly co-exist with one of the thyroid, but this last affords a much more rational explanation of the symptoms. Thymic enlargement may induce dyspnœa, but not the laryngeal disturbance. Atelectasis, too, the author believes, is often due to the impediment caused by this enlarged thyroid.

In a disease so rapid in its progress, little time remains for treatment ; but, where this is obtainable, leeches should be applied, and an emetic given. Where the hypertrophy is less considerable, and the disease more prolonged, the internal and external use of iodine would be deserving of a trial.—*London Med. Times, from Henle and Pfeufer's Zeitschrift.*

